# *N*′-[(2*Z*)-3-Allyl-4-oxo-1,3-thia­zolidin-2-yl­idene]-5-fluoro-3-phenyl-1*H*-indole-2-carbohydrazide

**DOI:** 10.1107/S1600536809012677

**Published:** 2009-04-08

**Authors:** Mehmet Akkurt, Selvi Karaca, Gökçe Cihan, Gültaze Çapan, Orhan Büyükgüngör

**Affiliations:** a Department of Physics, Faculty of Arts and Sciences, Erciyes University, 38039 Kayseri, Turkey; b Department of Pharmaceutical Chemistry, Faculty of Pharmacy, University of Istanbul, 34116 Beyazıt, Istanbul, Turkey; c Department of Physics, Faculty of Arts and Sciences, Ondokuz Mayıs University, 55139 Samsun, Turkey

## Abstract

In the title compound, C_21_H_17_FN_4_O_2_S, the planar indole fused-ring [maximum deviation 0.009 (1) Å] makes dihedral angles of 54.75 (9) and 14.90 (9)°, respectively, with the phenyl ring and the dihydro­thia­zolyl ring. The –CH2CH=CH_2_ substituent is disordered over two positions in a 0.51 (1):0.49 (1) ratio. An intra­molecular N—H⋯S hydrogen bond generates an *S*(5) ring motif. The two independent mol­ecules are linked into a dimer by two N—H⋯O hydrogen bonds, forming an *R*_2_^2^(10) ring motif. The crystal structure features inter­molecular C—H⋯π and π–π stacking [centroid–centroid distance = 3.679 (1) Å] inter­actions. C—H⋯O and C—H⋯F inter­actions are also present.

## Related literature

For the bactericidal, fungicidal, antitubercular and anticancer properties of 4-thia­zolidinone derivatives, see: Bonde & Gaikwad (2004 ▶); Güzel *et al.* (2006 ▶); Küçükgüzel *et al.* (2002 ▶); Kline *et al.* (2008 ▶); Ottanà *et al.* (2005 ▶); Ulusoy (2002 ▶); Zhou *et al.* (2008 ▶); Çapan *et al.* (1999 ▶).
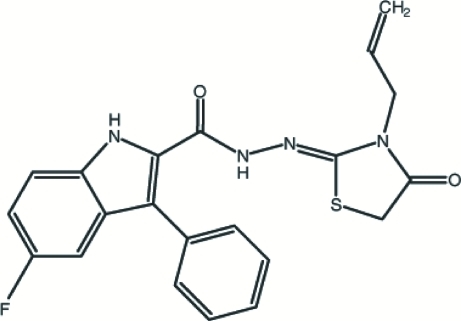


## Experimental

### 

#### Crystal data

C_21_H_17_FN_4_O_2_S
*M* *_r_* = 408.46
Monoclinic, 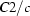

*a* = 21.9754 (6) Å
*b* = 14.7215 (5) Å
*c* = 16.2447 (4) Å
β = 132.022 (2)°
*V* = 3904.1 (2) Å^3^
*Z* = 8
Mo *K*α radiation
μ = 0.20 mm^−1^
*T* = 296 K
0.48 × 0.45 × 0.41 mm

#### Data collection

Stoe IPDS2 diffractometer
Absorption correction: integration (*X-RED32*; Stoe & Cie, 2002 ▶) *T*_min_ = 0.910, *T*_max_ = 0.922
27187 measured reflections
4444 independent reflections
3438 reflections with *I* > 2σ(*I*)
*R*_int_ = 0.031

#### Refinement

*R*[*F* ^2^ > 2σ(*F* ^2^)] = 0.040
*wR*(*F* ^2^) = 0.111
*S* = 1.04
4444 reflections
302 parameters
4 restraints
H atoms treated by a mixture of independent and constrained refinement
Δρ_max_ = 0.21 e Å^−3^
Δρ_min_ = −0.18 e Å^−3^

### 

Data collection: *X-AREA* (Stoe & Cie, 2002 ▶); cell refinement: *X-AREA*; data reduction: *X-RED32* (Stoe & Cie, 2002 ▶); program(s) used to solve structure: *SHELXS97* (Sheldrick, 2008 ▶); program(s) used to refine structure: *SHELXL97* (Sheldrick, 2008 ▶); molecular graphics: *ORTEP-3* (Farrugia, 1997 ▶); software used to prepare material for publication: *WinGX* (Farrugia, 1999 ▶).

## Supplementary Material

Crystal structure: contains datablocks global, I. DOI: 10.1107/S1600536809012677/ng2568sup1.cif

Structure factors: contains datablocks I. DOI: 10.1107/S1600536809012677/ng2568Isup2.hkl

Additional supplementary materials:  crystallographic information; 3D view; checkCIF report

## Figures and Tables

**Table 1:** Hydrogen-bond geometry (Å, °)

*D*—H⋯*A*	*D*—H	H⋯*A*	*D*⋯*A*	*D*—H⋯*A*
N1—H1⋯O1^i^	0.86	1.96	2.789 (2)	161
N2—H2*A*⋯S1	0.86	2.52	2.925 (2)	110
C17—H17*A*⋯O2^ii^	0.97	2.48	3.336 (3)	147
C20*B*—H20*B*⋯F1^iii^	0.93	2.37	3.284 (10)	168
C14—H14⋯*Cg*2^iii^	0.93	2.66	3.371 (2)	134

Symmetry codes: (i) 
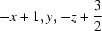
; (ii) 
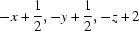
; (iii) 
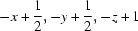
. *Cg*2 is the centroid of the N1/C1/C6–C8 ring.

**Fig. 1.**
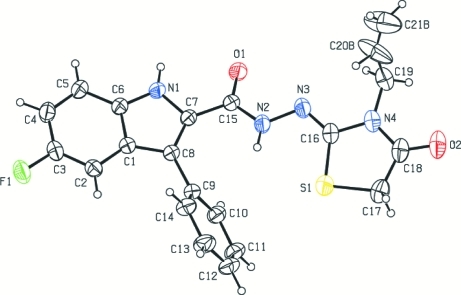
View of the title molecule with the atom-numbering scheme and 30% probability displacement ellipsoids. Only the major occupancy component of the disorder part is depicted.

**Fig. 2.**
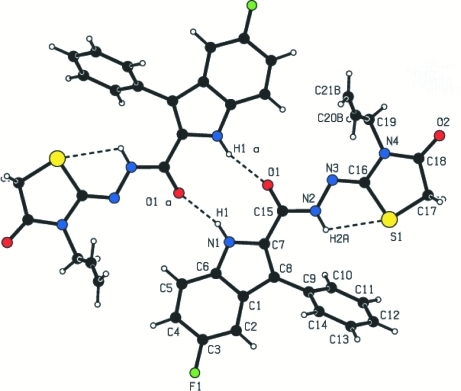
View of the two molecules linked into a dimer by two N—H···O hydrogen bonds [Symmetry code: (a) -1/2 + x,1/2 - y, -1/2 + z].

## supplementary crystallographic information

### Comment

Efforts to design, synthesize and screen new molecules that would mimic the actions of currently available chemotherapeutics have resulted in numerous promising candidates incorporating the 4-thiazolidinone system. Many 4-thiazolidinone derivatives have been shown to exhibit bactericidal (Bonde & Gaikwad, 2004; Kline *et al.*, 2008), fungicidal (Çapan *et al.*, 1999) antitubercular (Ulusoy, 2002; Küçükgüzel *et al.*, 2002; Güzel *et al.*, 2006) and anticancer (Zhou *et al.*, 2008) properties. Furthermore the structure of 4-thiazolidinones obtained from asymmetric thiourea derivatives has been frequently discussed due to the formation of regio-isomers involving 2- and 3-positions of the thiazolidinone ring depending upon the relative nucleophilic strengths of the thioamide N atoms (Ottanà *et al.*, 2005; Kline *et al.*, 2008). The nitrogen involved in ene-thiolization (*R*_1_N_1_=CSH—N_2_H*R*_2_/*R*_1_N_1_HCSH=N_2_*R*_2_) determines the regiochemical outcome of the cyclization. In this context, the title compound (2) was prepared from a thiosemicarbazide precursor (1) which may be regarded as an asymmetric thiourea analogue in an attempt to obtain a new molecule with antimicrobial action and to establish its definite structure. Thus spectroscopic and X-ray diffraction studies were carried out on (2) to determine the position of the 5-fluoro-3-phenyl-2-indolylcarbonylamino residue and the geometry about the C=N double bond.

In the title compound, (2), (Fig. 1), 1*H*-indole ring is essentially planar, with a maximum deviation of -0.009 (1) Å for C8. The nine-membered indole ring makes dihedral angles of 54.75 (9) and 14.90 (9) °, respectively, with the phenyl ring (C9–C14) and the 2,5-dihydro-1,3-thiazole ring (S1/N4/C16–C18). The dihedral angle between the (C9–C14) and (S1/N4/C16–C18) rings is 69.15 (9)°.

In the molecule, intramolecular N—H···S hydrogen bonding interactions generate *S*(5) ring motifs. In the crystal, the two independent molecules are linked into a dimer by two N—H···O hydrogen bonds, forming a *R*_2_^2^(10) ring motif (Fig. 2). The crystal structure, is further stabilized by intermolecular C—H···π [*Cg*1 and *Cg*2 are centroids of the S1/N4/C16–C18 and N1/C1/C6–C8 rings, respectively (Table 1)] and π–π interactions [*Cg*1···*Cg*2(*x*, -*y*, 1/2 + *z*) = 3.6791 (10) Å].

### Experimental

A mixture of 4-allyl-1-[(5-fluoro-3-phenyl-1*H*-indol-2-yl)carbonyl]-3-\ thiosemicarbazide (1) (0.0025 mol), ethyl bromoacetate (0.0025 mol) and fused sodium acetate (0.01 mol) in absolute ethanol (15 ml) was heated under reflux for 3 h. The solid thus obtained (2) was filtered, dried and purified by recrystallization from a mixture of ethanol: chloroform [Yield: 63.7%, m.p.: 535–538 K]. IR (KBr) ν = 3309, 3247 (N—H), 1716 (C=O), 1654 (C=O), 1608 (C=N) cm^-1^; ^1^H-NMR (DMSO-d_6_, 500 MHz) δ = 4.05 (2*H*, s, S—CH_2_), 4.24 (2*H*, s*, N—CH_2_CH=CH_2_), 5.12 (2*H*, s*, N—CH_2_CH=CH_2_), 5.81 (1*H*, s*, N—CH_2_CH=CH_2_), 7.11 (1*H*, dt, J = 9.1, 2.4 Hz, H6-indole), 7.15 (1*H*, d*, J = 9.3 Hz, H4-indole), 7.36 (1*H*, s*, 3-C_6_H_5_ (H4)-indole), 7.49–7.46 (5*H*, m, H7, 3-C_6_H_5_ (H2, H6, H3, H5)-indole), 9.78 (1*H*, s, CONH), 11.87 (1*H*, s, NH-indole) p.p.m. (* = broad). Analysis calculated for C_21_H_17_FN_4_O_2_S: C 61.75, H 4.20, N 13.72%. Found: C 61.84, H4.87, N 13.69%.

### Refinement

The two H atoms of the C19 atom were found from a difference Fourier map and refined freely. The rest H atoms were positioned geometrically and refined a riding model, with N—H = 0.86, C—H = 0.93 and 0.97 Å, and with *U*_iso_(H) = 1.2*U*_eq_(C,*N*). The site-occupation factors of the disordered atoms refined to 0.487 (13) for C20A and C21A and 0.513 (13) for C20B and C21B.

### Crystal data

**Table:**

C_21_H_17_FN_4_O_2_S	*F*(000) = 1696
*M**_r_* = 408.46	*D*_x_ = 1.390 Mg m^−^^3^
Monoclinic, *C*2/*c*	Mo *K*α radiation, λ = 0.71073 Å
Hall symbol: -C 2yc	Cell parameters from 28593 reflections
*a* = 21.9754 (6) Å	θ = 1.7–28.0°
*b* = 14.7215 (5) Å	µ = 0.20 mm^−^^1^
*c* = 16.2447 (4) Å	*T* = 296 K
β = 132.022 (2)°	Prism, colourless
*V* = 3904.1 (2) Å^3^	0.48 × 0.45 × 0.41 mm
*Z* = 8	

### Data collection

**Table:**

STOE IPDS2 diffractometer	4444 independent reflections
Radiation source: sealed X-ray tube, 12 x 0.4 mm long-fine focus	3438 reflections with *I* > 2σ(*I*)
plane graphite	*R*_int_ = 0.031
Detector resolution: 6.67 pixels mm^-1^	θ_max_ = 27.6°, θ_min_ = 1.9°
ω scans	*h* = −28→28
Absorption correction: integration (*X-RED32*; Stoe & Cie, 2002)	*k* = −19→19
*T*_min_ = 0.910, *T*_max_ = 0.922	*l* = −21→21
27187 measured reflections	

### Refinement

**Table:**

Refinement on *F*^2^	Secondary atom site location: difference Fourier map
Least-squares matrix: full	Hydrogen site location: inferred from neighbouring sites
*R*[*F*^2^ > 2σ(*F*^2^)] = 0.040	H atoms treated by a mixture of independent and constrained refinement
*wR*(*F*^2^) = 0.111	*w* = 1/[σ^2^(*F*_o_^2^) + (0.0574*P*)^2^ + 0.7834*P*] where *P* = (*F*_o_^2^ + 2*F*_c_^2^)/3
*S* = 1.04	(Δ/σ)_max_ = 0.001
4444 reflections	Δρ_max_ = 0.21 e Å^−^^3^
302 parameters	Δρ_min_ = −0.17 e Å^−^^3^
4 restraints	Extinction correction: *SHELXL*, FC^*^=KFC[1+0.001XFC^2^Λ^3^/SIN(2Θ)]^-1/4^
Primary atom site location: structure-invariant direct methods	Extinction coefficient: 0.0009 (3)

### Special details

**Table:**

Geometry. Bond distances, angles *etc*. have been calculated using the rounded fractional coordinates. All su's are estimated from the variances of the (full) variance-covariance matrix. The cell e.s.d.'s are taken into account in the estimation of distances, angles and torsion angles
Refinement. Refinement on *F*^2^ for ALL reflections except those flagged by the user for potential systematic errors. Weighted *R*-factors *wR* and all goodnesses of fit *S* are based on *F*^2^, conventional *R*-factors *R* are based on *F*, with *F* set to zero for negative *F*^2^. The observed criterion of *F*^2^ > σ(*F*^2^) is used only for calculating -*R*-factor-obs *etc*. and is not relevant to the choice of reflections for refinement. *R*-factors based on *F*^2^ are statistically about twice as large as those based on *F*, and *R*-factors based on ALL data will be even larger.

### Fractional atomic coordinates and isotropic or equivalent isotropic displacement parameters (Å^2^)

**Table:**

	*x*	*y*	*z*	*U*_iso_*/*U*_eq_	Occ. (<1)
S1	0.21369 (3)	0.12252 (4)	0.77932 (4)	0.0740 (2)	
F1	0.17462 (7)	0.15524 (8)	0.14694 (8)	0.0831 (4)	
O1	0.44729 (7)	0.05930 (11)	0.79608 (9)	0.0802 (5)	
O2	0.31157 (12)	0.14689 (11)	1.08254 (13)	0.0975 (7)	
N1	0.38666 (7)	0.08425 (9)	0.58683 (10)	0.0539 (4)	
N2	0.33122 (8)	0.10877 (10)	0.75039 (10)	0.0607 (4)	
N3	0.36940 (9)	0.11650 (10)	0.86108 (11)	0.0640 (5)	
N4	0.35123 (10)	0.13330 (10)	0.98479 (12)	0.0683 (5)	
C1	0.26040 (8)	0.11360 (10)	0.42564 (12)	0.0500 (4)	
C2	0.20191 (9)	0.13246 (11)	0.31160 (13)	0.0572 (5)	
C3	0.22958 (11)	0.13647 (12)	0.25772 (13)	0.0616 (5)	
C4	0.31034 (11)	0.12236 (12)	0.30720 (14)	0.0650 (6)	
C5	0.36848 (10)	0.10389 (12)	0.41841 (14)	0.0616 (5)	
C6	0.34253 (9)	0.09967 (10)	0.47696 (12)	0.0516 (4)	
C7	0.33549 (8)	0.08846 (10)	0.60763 (12)	0.0502 (4)	
C8	0.25619 (8)	0.10495 (10)	0.50956 (12)	0.0482 (4)	
C9	0.17981 (8)	0.10843 (10)	0.48911 (12)	0.0504 (4)	
C10	0.15738 (10)	0.03640 (12)	0.51928 (14)	0.0632 (5)	
C11	0.08451 (11)	0.03946 (15)	0.49644 (16)	0.0776 (7)	
C12	0.03309 (11)	0.11353 (16)	0.44348 (17)	0.0802 (7)	
C13	0.05436 (10)	0.18427 (15)	0.41220 (17)	0.0762 (7)	
C14	0.12698 (10)	0.18184 (12)	0.43468 (15)	0.0627 (5)	
C15	0.37596 (8)	0.08369 (11)	0.72532 (12)	0.0535 (5)	
C16	0.32070 (11)	0.12386 (11)	0.87750 (13)	0.0594 (5)	
C17	0.20908 (14)	0.13479 (16)	0.88526 (19)	0.0822 (8)	
C18	0.29483 (14)	0.13926 (13)	0.99488 (17)	0.0741 (7)	
C19	0.43942 (16)	0.13472 (19)	1.08020 (18)	0.0892 (9)	
C20B	0.4660 (5)	0.2330 (6)	1.0835 (8)	0.146 (3)	0.513 (13)
C21B	0.5130 (6)	0.2848 (8)	1.1365 (7)	0.166 (4)	0.513 (13)
C21A	0.4594 (7)	0.2904 (7)	1.0986 (7)	0.105 (3)	0.487 (13)
C20A	0.4920 (5)	0.2117 (6)	1.1294 (7)	0.106 (3)	0.487 (13)
H2	0.14700	0.14170	0.27480	0.059 (4)*	
H1	0.43840	0.07360	0.63550	0.061 (5)*	
H5	0.42300	0.09450	0.45360	0.069 (5)*	
H10	0.19150	−0.01390	0.55490	0.072 (5)*	
H11	0.06990	−0.00890	0.51700	0.098 (7)*	
H12	−0.01560	0.11540	0.42910	0.097 (7)*	
H13	0.01970	0.23410	0.37570	0.103 (7)*	
H14	0.14080	0.23020	0.41300	0.069 (5)*	
H17A	0.17970	0.18980	0.87300	0.112 (8)*	
H17B	0.18060	0.08350	0.88360	0.099 (7)*	
H19A	0.4441 (17)	0.1087 (19)	1.132 (2)	0.113 (9)*	
H19B	0.4668 (18)	0.086 (2)	1.066 (2)	0.132 (10)*	
H20B	0.42670	0.25770	1.01300	0.1750*	0.513 (13)
H21C	0.55760	0.27220	1.21060	0.1990*	0.513 (13)
H21D	0.50830	0.34150	1.10740	0.1990*	0.513 (13)
H2A	0.27970	0.11990	0.69870	0.086 (6)*	
H4	0.32490	0.12540	0.26510	0.076 (6)*	
H20A	0.54860	0.20480	1.18250	0.1270*	0.487 (13)
H21A	0.40270	0.29620	1.04540	0.1260*	0.487 (13)
H21B	0.49230	0.34190	1.12940	0.1260*	0.487 (13)

### Atomic displacement parameters (Å^2^)

**Table:**

	*U*^11^	*U*^22^	*U*^33^	*U*^12^	*U*^13^	*U*^23^
S1	0.0723 (3)	0.0928 (4)	0.0657 (3)	0.0010 (2)	0.0498 (2)	−0.0014 (2)
F1	0.0882 (7)	0.1037 (8)	0.0487 (5)	0.0056 (6)	0.0422 (5)	0.0057 (5)
O1	0.0452 (6)	0.1385 (12)	0.0528 (6)	0.0108 (6)	0.0311 (5)	0.0155 (7)
O2	0.1405 (14)	0.1047 (11)	0.0858 (10)	0.0000 (10)	0.0916 (11)	−0.0059 (8)
N1	0.0420 (6)	0.0715 (8)	0.0493 (6)	0.0044 (5)	0.0310 (5)	0.0049 (6)
N2	0.0526 (7)	0.0862 (9)	0.0456 (6)	0.0083 (6)	0.0338 (6)	0.0055 (6)
N3	0.0659 (8)	0.0787 (9)	0.0473 (7)	0.0029 (7)	0.0379 (6)	0.0020 (6)
N4	0.0846 (10)	0.0724 (9)	0.0552 (8)	0.0019 (7)	0.0498 (8)	−0.0001 (6)
C1	0.0472 (7)	0.0554 (8)	0.0474 (7)	−0.0009 (6)	0.0317 (6)	−0.0003 (6)
C2	0.0520 (8)	0.0645 (9)	0.0481 (8)	0.0010 (6)	0.0306 (7)	−0.0003 (6)
C3	0.0692 (10)	0.0656 (10)	0.0458 (8)	−0.0002 (7)	0.0368 (8)	−0.0001 (7)
C4	0.0769 (10)	0.0737 (11)	0.0626 (10)	−0.0028 (8)	0.0542 (9)	−0.0028 (8)
C5	0.0610 (9)	0.0743 (10)	0.0642 (9)	0.0008 (7)	0.0480 (8)	0.0004 (8)
C6	0.0497 (7)	0.0585 (8)	0.0511 (7)	0.0010 (6)	0.0356 (6)	0.0005 (6)
C7	0.0460 (7)	0.0589 (8)	0.0510 (7)	0.0003 (6)	0.0347 (6)	0.0030 (6)
C8	0.0438 (6)	0.0548 (8)	0.0475 (7)	−0.0017 (5)	0.0312 (6)	0.0004 (6)
C9	0.0421 (7)	0.0614 (8)	0.0469 (7)	−0.0042 (6)	0.0295 (6)	−0.0026 (6)
C10	0.0545 (8)	0.0744 (11)	0.0623 (9)	−0.0029 (7)	0.0397 (8)	0.0082 (8)
C11	0.0587 (9)	0.1023 (14)	0.0775 (11)	−0.0106 (9)	0.0479 (9)	0.0122 (10)
C12	0.0487 (8)	0.1175 (16)	0.0786 (12)	−0.0027 (9)	0.0443 (9)	0.0071 (11)
C13	0.0537 (9)	0.0897 (13)	0.0840 (13)	0.0115 (8)	0.0456 (9)	0.0106 (10)
C14	0.0527 (8)	0.0656 (10)	0.0720 (10)	0.0006 (7)	0.0427 (8)	0.0041 (8)
C15	0.0456 (7)	0.0660 (9)	0.0496 (8)	−0.0036 (6)	0.0322 (7)	0.0023 (7)
C16	0.0733 (10)	0.0588 (9)	0.0541 (8)	0.0036 (7)	0.0459 (8)	0.0019 (7)
C17	0.0978 (14)	0.0875 (14)	0.0915 (14)	0.0088 (11)	0.0758 (13)	0.0004 (11)
C18	0.1055 (14)	0.0660 (11)	0.0765 (12)	0.0041 (9)	0.0715 (12)	0.0002 (8)
C19	0.0908 (15)	0.1127 (19)	0.0540 (11)	−0.0049 (13)	0.0443 (11)	−0.0017 (11)
C20B	0.095 (5)	0.109 (6)	0.070 (5)	−0.011 (4)	−0.012 (4)	0.013 (4)
C21B	0.093 (6)	0.140 (7)	0.150 (7)	−0.032 (5)	0.034 (5)	0.020 (5)
C21A	0.106 (6)	0.115 (6)	0.075 (4)	0.005 (4)	0.053 (4)	0.009 (4)
C20A	0.074 (4)	0.138 (6)	0.054 (4)	0.004 (4)	0.021 (3)	0.003 (4)

### Geometric parameters (Å, °)

**Table:**

S1—C16	1.747 (2)	C10—C11	1.382 (4)
S1—C17	1.800 (3)	C11—C12	1.380 (3)
F1—C3	1.3652 (19)	C12—C13	1.371 (4)
O1—C15	1.223 (2)	C13—C14	1.379 (4)
O2—C18	1.214 (3)	C17—C18	1.495 (4)
N1—C6	1.3639 (19)	C19—C20B	1.548 (10)
N1—C7	1.375 (3)	C19—C20A	1.422 (10)
N2—N3	1.3894 (19)	C20A—C21A	1.275 (14)
N2—C15	1.344 (3)	C20B—C21B	1.095 (15)
N3—C16	1.269 (4)	C2—H2	0.9300
N4—C16	1.393 (2)	C4—H4	0.9300
N4—C18	1.358 (4)	C5—H5	0.9300
N4—C19	1.462 (4)	C10—H10	0.9300
N1—H1	0.8600	C11—H11	0.9300
N2—H2A	0.8600	C12—H12	0.9300
C1—C6	1.407 (3)	C13—H13	0.9300
C1—C8	1.433 (3)	C14—H14	0.9300
C1—C2	1.405 (2)	C17—H17A	0.9700
C2—C3	1.361 (3)	C17—H17B	0.9700
C3—C4	1.389 (4)	C19—H19A	0.87 (3)
C4—C5	1.371 (2)	C19—H19B	1.06 (4)
C5—C6	1.400 (3)	C20A—H20A	0.9300
C7—C8	1.385 (2)	C20B—H20B	0.9300
C7—C15	1.475 (2)	C21A—H21A	0.9300
C8—C9	1.478 (3)	C21A—H21B	0.9300
C9—C10	1.390 (3)	C21B—H21C	0.9300
C9—C14	1.387 (3)	C21B—H21D	0.9300
			
S1···N2	2.925 (2)	C21B···S1^ix^	3.605 (13)
S1···C11	3.634 (2)	C1···H14	3.0300
S1···C21B^i^	3.605 (13)	C1···H14^iii^	3.0400
S1···H2A	2.5200	C2···H14	3.0900
F1···C10^ii^	3.369 (2)	C6···H14^iii^	2.9500
F1···C20B^iii^	3.284 (10)	C7···H14^iii^	2.7800
F1···C16^iii^	3.286 (2)	C7···H10	3.0600
F1···C21A^iii^	3.082 (9)	C8···H2A	2.7600
F1···H11^ii^	2.8100	C8···H14^iii^	2.9600
F1···H10^ii^	2.7300	C9···H2	3.0900
F1···H20B^iii^	2.3700	C9···H2A	2.5400
F1···H21A^iii^	2.4600	C10···H2A	2.6000
O1···N1	2.7205 (18)	C14···H2	2.9700
O1···N3	2.678 (3)	C15···H1^iv^	3.0700
O1···N1^iv^	2.789 (2)	C16···H20B	2.7000
O2···C17^v^	3.336 (3)	C17···H21C^i^	2.8900
O1···H1^iv^	1.9600	C18···H21A	3.0000
O1···H1	2.4900	C21B···H17A^ix^	3.0800
O1···H19B^vi^	2.74 (3)	H1···O1^iv^	1.9600
O2···H4^vii^	2.8000	H1···O1	2.4900
O2···H19A	2.51 (4)	H1···C15^iv^	3.0700
O2···H17A^v^	2.4800	H2···C14	2.9700
N1···O1	2.7205 (18)	H2···C9	3.0900
N1···O1^iv^	2.789 (2)	H2···H12^x^	2.5800
N2···C10	3.259 (2)	H2A···C9	2.5400
N2···S1	2.925 (2)	H2A···C10	2.6000
N2···C9	3.1904 (19)	H2A···C8	2.7600
N3···O1	2.678 (3)	H2A···S1	2.5200
N3···C20B	3.199 (10)	H4···O2^xi^	2.8000
N3···C5^viii^	3.379 (2)	H4···H20A^iv^	2.5800
N4···C6^viii^	3.433 (2)	H10···F1^viii^	2.7300
N1···H14^iii^	2.8000	H10···C7	3.0600
N3···H19B	2.52 (2)	H11···F1^viii^	2.8100
N3···H20B	2.8000	H11···H12^xii^	2.4600
N4···H21A	2.5500	H12···H11^xii^	2.4600
C1···C14^iii^	3.581 (2)	H12···H2^x^	2.5800
C2···C14	3.413 (3)	H14···N1^iii^	2.8000
C5···N3^ii^	3.379 (2)	H14···C1	3.0300
C5···C16^ii^	3.443 (2)	H14···C2	3.0900
C6···C14^iii^	3.401 (2)	H14···C7^iii^	2.7800
C6···N4^ii^	3.433 (2)	H14···C8^iii^	2.9600
C6···C16^ii^	3.554 (2)	H14···C1^iii^	3.0400
C9···N2	3.1904 (19)	H14···C6^iii^	2.9500
C10···N2	3.259 (2)	H17A···O2^v^	2.4800
C10···F1^viii^	3.369 (2)	H17A···C21B^i^	3.0800
C11···S1	3.634 (2)	H17A···H21C^i^	2.2300
C14···C2	3.413 (3)	H19A···O2	2.51 (4)
C14···C6^iii^	3.401 (2)	H19B···O1^vi^	2.74 (3)
C14···C1^iii^	3.581 (2)	H19B···N3	2.52 (2)
C16···C6^viii^	3.554 (2)	H20A···H4^iv^	2.5800
C16···F1^iii^	3.286 (2)	H20B···C16	2.7000
C16···C5^viii^	3.443 (2)	H20B···N3	2.8000
C17···O2^v^	3.336 (3)	H20B···F1^iii^	2.3700
C18···C21A	3.565 (14)	H21A···F1^iii^	2.4600
C20B···N3	3.199 (10)	H21A···N4	2.5500
C20B···F1^iii^	3.284 (10)	H21A···C18	3.0000
C21A···C18	3.565 (14)	H21C···H17A^ix^	2.2300
C21A···F1^iii^	3.082 (9)	H21C···C17^ix^	2.8900
			
C16—S1—C17	91.66 (12)	O2—C18—C17	123.5 (3)
C6—N1—C7	109.39 (16)	N4—C18—C17	112.2 (2)
N3—N2—C15	118.97 (16)	N4—C19—C20A	127.5 (4)
N2—N3—C16	114.55 (17)	N4—C19—C20B	104.6 (4)
C16—N4—C18	116.3 (2)	C19—C20A—C21A	118.2 (10)
C16—N4—C19	120.9 (2)	C19—C20B—C21B	144.5 (10)
C18—N4—C19	122.7 (2)	C1—C2—H2	122.00
C7—N1—H1	125.00	C3—C2—H2	122.00
C6—N1—H1	125.00	C3—C4—H4	120.00
N3—N2—H2A	120.00	C5—C4—H4	120.00
C15—N2—H2A	121.00	C4—C5—H5	121.00
C2—C1—C6	119.25 (19)	C6—C5—H5	121.00
C2—C1—C8	133.4 (2)	C9—C10—H10	120.00
C6—C1—C8	107.31 (14)	C11—C10—H10	120.00
C1—C2—C3	116.7 (2)	C10—C11—H11	120.00
F1—C3—C2	118.3 (2)	C12—C11—H11	120.00
C2—C3—C4	124.70 (16)	C11—C12—H12	120.00
F1—C3—C4	117.0 (2)	C13—C12—H12	120.00
C3—C4—C5	119.7 (2)	C12—C13—H13	120.00
C4—C5—C6	117.4 (2)	C14—C13—H13	120.00
C1—C6—C5	122.36 (15)	C9—C14—H14	119.00
N1—C6—C1	107.82 (18)	C13—C14—H14	120.00
N1—C6—C5	129.8 (2)	S1—C17—H17A	110.00
C8—C7—C15	134.6 (2)	S1—C17—H17B	110.00
N1—C7—C15	115.70 (16)	C18—C17—H17A	110.00
N1—C7—C8	109.36 (15)	C18—C17—H17B	110.00
C1—C8—C9	124.76 (14)	H17A—C17—H17B	108.00
C7—C8—C9	129.06 (16)	N4—C19—H19A	104 (2)
C1—C8—C7	106.10 (18)	N4—C19—H19B	107.7 (16)
C8—C9—C10	120.79 (16)	C20B—C19—H19A	125.7 (19)
C8—C9—C14	120.69 (17)	C20B—C19—H19B	113 (2)
C10—C9—C14	118.5 (2)	H19A—C19—H19B	101 (3)
C9—C10—C11	120.10 (18)	C20A—C19—H19A	106.5 (19)
C10—C11—C12	120.8 (2)	C20A—C19—H19B	107 (2)
C11—C12—C13	119.4 (3)	C21A—C20A—H20A	121.00
C12—C13—C14	120.2 (2)	C19—C20A—H20A	121.00
C9—C14—C13	121.0 (2)	C21B—C20B—H20B	108.00
N2—C15—C7	116.83 (16)	C19—C20B—H20B	108.00
O1—C15—N2	122.24 (15)	C20A—C21A—H21A	120.00
O1—C15—C7	120.92 (19)	H21A—C21A—H21B	120.00
S1—C16—N4	111.7 (2)	C20A—C21A—H21B	120.00
N3—C16—N4	120.3 (2)	C20B—C21B—H21D	120.00
S1—C16—N3	128.04 (13)	H21C—C21B—H21D	120.00
S1—C17—C18	108.1 (2)	C20B—C21B—H21C	120.00
O2—C18—N4	124.3 (3)		
			
C17—S1—C16—N4	−0.12 (14)	C2—C1—C6—C5	0.0 (2)
C17—S1—C16—N3	179.05 (17)	C1—C2—C3—F1	−179.58 (14)
C16—S1—C17—C18	−0.04 (16)	C1—C2—C3—C4	0.7 (3)
C6—N1—C7—C8	1.30 (17)	C2—C3—C4—C5	−0.8 (3)
C7—N1—C6—C5	178.52 (16)	F1—C3—C4—C5	179.53 (16)
C7—N1—C6—C1	−0.40 (17)	C3—C4—C5—C6	0.4 (3)
C6—N1—C7—C15	−173.18 (13)	C4—C5—C6—N1	−178.79 (16)
N3—N2—C15—O1	6.9 (3)	C4—C5—C6—C1	0.0 (2)
C15—N2—N3—C16	−167.60 (16)	C8—C7—C15—N2	−7.9 (3)
N3—N2—C15—C7	−171.95 (14)	N1—C7—C8—C9	175.12 (14)
N2—N3—C16—N4	−179.16 (14)	C15—C7—C8—C9	−11.9 (3)
N2—N3—C16—S1	1.7 (2)	C8—C7—C15—O1	173.20 (18)
C16—N4—C18—O2	179.34 (18)	C15—C7—C8—C1	171.37 (17)
C19—N4—C18—O2	0.5 (3)	N1—C7—C8—C1	−1.63 (17)
C18—N4—C16—S1	0.27 (19)	N1—C7—C15—N2	164.77 (15)
C18—N4—C19—C20B	−100.8 (5)	N1—C7—C15—O1	−14.1 (2)
C19—N4—C16—S1	179.10 (16)	C1—C8—C9—C10	122.17 (17)
C18—N4—C16—N3	−178.97 (16)	C1—C8—C9—C14	−54.8 (2)
C19—N4—C16—N3	−0.1 (3)	C7—C8—C9—C10	−54.0 (2)
C16—N4—C19—C20B	80.4 (5)	C7—C8—C9—C14	129.01 (18)
C16—N4—C18—C17	−0.3 (2)	C8—C9—C10—C11	−177.95 (16)
C19—N4—C18—C17	−179.11 (19)	C10—C9—C14—C13	0.9 (3)
C6—C1—C2—C3	−0.3 (2)	C14—C9—C10—C11	−0.9 (3)
C8—C1—C2—C3	179.17 (17)	C8—C9—C14—C13	177.94 (16)
C2—C1—C6—N1	179.01 (14)	C9—C10—C11—C12	0.1 (3)
C2—C1—C8—C9	4.9 (3)	C10—C11—C12—C13	0.8 (3)
C2—C1—C8—C7	−178.18 (17)	C11—C12—C13—C14	−0.8 (3)
C6—C1—C8—C7	1.37 (17)	C12—C13—C14—C9	−0.1 (3)
C6—C1—C8—C9	−175.56 (14)	S1—C17—C18—N4	0.2 (2)
C8—C1—C6—N1	−0.62 (17)	S1—C17—C18—O2	−179.46 (17)
C8—C1—C6—C5	−179.63 (15)	N4—C19—C20B—C21B	158 (2)

Symmetry codes: (i) *x*−1/2, −*y*+1/2, *z*−1/2; (ii) *x*, −*y*, *z*−1/2; (iii) −*x*+1/2, −*y*+1/2, −*z*+1; (iv) −*x*+1, *y*, −*z*+3/2; (v) −*x*+1/2, −*y*+1/2, −*z*+2; (vi) −*x*+1, −*y*, −*z*+2; (vii) *x*, *y*, *z*+1; (viii) *x*, −*y*, *z*+1/2; (ix) *x*+1/2, −*y*+1/2, *z*+1/2; (x) −*x*, *y*, −*z*+1/2; (xi) *x*, *y*, *z*−1; (xii) −*x*, −*y*, −*z*+1.

### Hydrogen-bond geometry (Å, °)

**Table:**

*D*—H···*A*	*D*—H	H···*A*	*D*···*A*	*D*—H···*A*
N1—H1···O1^iv^	0.86	1.96	2.789 (2)	161
N2—H2A···S1	0.86	2.52	2.925 (2)	110
C17—H17A···O2^v^	0.97	2.48	3.336 (3)	147
C19—H19A···O2	0.87 (3)	2.51 (4)	2.841 (5)	103 (3)
C20B—H20B···F1^iii^	0.93	2.37	3.284 (10)	168
C14—H14···Cg2^iii^	0.93	2.66	3.371 (2)	134

Symmetry codes: (iv) −*x*+1, *y*, −*z*+3/2; (v) −*x*+1/2, −*y*+1/2, −*z*+2; (iii) −*x*+1/2, −*y*+1/2, −*z*+1.
